# Cerebellar ataxia with sensory ganglionopathy; does autoimmunity have a role to play?

**DOI:** 10.1186/s40673-017-0079-1

**Published:** 2017-12-22

**Authors:** Panagiotis Zis, Ptolemaios Georgios Sarrigiannis, Dasappaiah Ganesh Rao, Nigel Hoggard, David Surendran Sanders, Marios Hadjivassiliou

**Affiliations:** 1 Academic Department of Neurosciences, Sheffield Teaching Hospitals NHS Foundation Trust, Sheffield, UK; 2 University of Sheffield, Sheffield, UK; 3 Department of Neuroradiology, Sheffield Teaching Hospitals NHS Foundaiton Trust, Sheffield, UK; 4 Academic Unit of Gastroenterology, Sheffield Teaching Hospitals NHS Foundation Trust, Sheffield, UK

**Keywords:** Ganglionopathy, Cerebellar ataxia, Gluten, Autoimmunity

## Abstract

**Background and purpose:**

Cerebellar ataxia with sensory ganglionopathy (SG) is a disabling combination of neurological dysfunction usually seen as part of some hereditary ataxias. However, patients may present with this combination without a genetic cause.

**Methods:**

We reviewed records of all patients that have been referred to the Sheffield Ataxia Centre who had neurophysiological and imaging data suggestive of SG and cerebellar ataxia respectively. We excluded patients with Friedreich’s ataxia, a common cause of this combination. All patients were screened for genetic causes and underwent extensive investigations.

**Results:**

We identified 40 patients (45% males, mean age at symptom onset 53.7 ± 14.7 years) with combined cerebellar ataxia and SG. The majority of patients (40%) were initially diagnosed with cerebellar dysfunction and 30% were initially diagnosed with SG. For 30% the two diagnoses were made at the same time. The mean latency between the two diagnoses was 6.5 ± 8.9 years (range 0–44). The commonest initial manifestation was unsteadiness (77.5%) followed by patchy sensory loss (17.5%) and peripheral neuropathic pain (5%).

Nineteen patients (47.5%) had gluten sensitivity, of whom 3 patients (7.5%) had biopsy proven coeliac disease. Other abnormal immunological tests were present in another 15 patients. Six patients had malignancy, which was diagnosed within 5 years of the neurological symptoms. Only 3 patients (7.5%) were classified as having a truly idiopathic combination of cerebellar ataxia with SG.

**Conclusion:**

Our case series highlights that amongst patients with the unusual combination of cerebellar ataxia and SG, immune pathogenesis plays a significant role.

## Introduction

Ataxia is a term used to describe unsteadiness and poor co-ordination of movements. It can occur secondary to cerebellar dysfunction (cerebellar ataxia) or because of dysfunction within the peripheral or central sensory pathways (sensory ataxia).

Cerebellar ataxia can be inherited (e.g. Friedreich’s ataxia and spinocerenellar ataxias, known as SCAs) or acquired (e.g. gluten ataxia and paraneoplastic) [1].

Sensory ataxia is commonly seen in neuropathies that involve sensory fibers and are more prominent in pure sensory neuropathies involving the dorsal root ganglia, commonly referred to as sensory ganglionopathies (SG) [2]. Causes of SG can be inherited (e.g. mitochondrial disorders) or acquired (e.g. paraneoplastic) [3–5].

Cerebellar ataxia combined with SG is a relatively rare neurological combination, which can sometimes be seen in the context of hereditary ataxias [2] (e.g. Friedreich’s ataxia and SCA4, mitochondrial disease and Cerebellar ataxia, neuropathy and vestibular areflexia syndrome (CANVAS) [6]) or as a result of exposure to toxins (e.g. amiodarone [7]).

We present a case series of patients with this unusual combination, in an attempt to shed light into possible underlying aetiology.

## Methods

### Standard protocol approvals, registrations, and patient consents

This is a retrospective observational case series of patients regularly attending the Ataxia clinic based at the Royal Hallamshire Hospital (Sheffield, UK). The South Yorkshire Research Ethics Committee has confirmed that no ethical approval is indicated given that all investigations were clinically indicated and did not form part of a research study.

### Patient selection

All recruited patients had clinical and neuroimaging evidence of progressive cerebellar dysfunction as well as clinical and neurophysiological evidence of SG.

### Neurophysiological assessments

All patients underwent detailed nerve conduction studies including median (sensory and motor), ulnar (sensory and motor), superficial radial (sensory), sural (sensory), superficial peroneal (sensory) and tibial (motor). All sensory nerve conduction studies were performed bilaterally. On all occasions supramaximal stimulation of the nerves was performed.

The diagnosis of SG was based on the established diagnostic criteria [8, 9]. Clinically patients that presented with signs and symptoms of patchy sensory symptoms and reduced or absent tendon reflexes underwent nerve conduction studies. SG was confirmed electrophysiologically with complete absence of sensory nerve action potentials (SNAPs) or asymmetrical sensory fiber involvement (asymmetrical SNAPs) with no motor involvement.

### Neuroimaging assessments

All patients underwent a magnetic resonance imaging (MRI) of the brain and a magnetic resonance spectroscopy (MRS) of the cerebellum. The latter technique is validated and is used to determine the presence of cerebellar dysfunction, even in the absence of cerebellar atrophy [10]. We measured the N-acetyl-aspartate/creatine (NAA/Cr) ratios in the vermis and the cerebellar hemispheres. Only patients with cerebellar atrophy and/or abnormal NAA/Cr ratios in either the vermis or the hemispheres and/or cerebellar atrophy in the MRI where considered as having cerebellar dysfunction.

### Serologic testing

Serological testing was performed and included extensive testing for possible acquired causes of cerebellar ataxia and sensory ganglionopathy. Human leukocyte antigen (HLA) typing was performed by the regional blood-transfusion service.

### Genetic testing

Patients with early onset cerebellar ataxia and/or family history of ataxia were tested genetically for possible genetic causes. A muscle biopsy was done in patients with a suspected mitochondrial disease. By default we did not include in this case series patients with Friedreich’s ataxia (FA) as this is a well known cause of this combination of neurological deficits.

### Enteropathy

Patients with gluten sensitivity underwent gastroscopy and duodenal biopsy to establish the presence of enteropathy. All biopsies were histologically assessed for evidence of enteropathy (triad of villous atrophy, crypt hyperplasia, and increase in intraepithelial lymphocytes).

### Statistical analysis

A database was developed using the statistical software package SPSS (version 23.0 for Macintosh). Descriptive statistics were examined for each variable.

## Results

### Clinical characteristics

We identified 40 patients (45% males) with combined cerebellar ataxia and sensory ganglionopathy. Mean age at onset of the symptoms was 53.7 ± 14.7 years (range 8–80) and mean age at first presentation was 56.6 ± 13.5 years (range 14–81). At presentation, the majority of patients (40%) were diagnosed with cerebellar ataxia and 30% were diagnosed with SG. For 30% the two diagnoses were made at the same time. The mean latency between the two diagnoses was 6.5 ± 8.9 years (range 0–44). The commonest initial manifestation was unsteadiness (77.5%) followed by patchy sensory loss (17.5%) and peripheral neuropathic pain (5%).

### Neurophysiology

Sixteen patients (40%) had absent SNAPs, whereas the rest had asymmetrical SNAPs. No patients had abnormal compound muscle action potentials (CMAPs). Table 1 summarizes the neurophysiological characteristics of a typical patient with asymmetrical SNAPs (Patient 1) and of a patient with completely absent SNAPs (Patient 2) from these series.

**Table 1:** Neurophysiological characteristics of a typical patient with asymmetrical SNAPs (Patient 1) and of a patient with completely absent SNAPs (Patient 2) from these series

Subject	NCS Parameter	Median CMAP	Ulnar (ADM) CMAP	Tibial CMAP	Median SNAP		Ulnar SNAP		Radial SNAP		Sural SNAP		Superficial Peroneal SNAP	
	Side	(R)	(R)	(R)	(R)	(L)	(R)	(L)	(R)	(L)	(R)	(L)	(R)	(L)
Patient 1 (Asymmetric SNAPs) 46Y, Female	Amplitude	14.1 mV	10.7 mV	9.2 mV	17.4 μV	6.1 μV	3.1 μV	5.7 μV	18.4 μV	12.4 μV	21.5 μV	12.8 μV	7.7 μV	3.4. μV
	Conduction velocity	60 m/s (Elbow-Wrist)	60 m/s (Below elbow – Wrist) 70 m/s (Above elbow – below elbow)	NC	56 m/s (Digit III –Wrist)	60 m/s (Digit III –Wrist)	58 m/s (Digit V –Wrist)	57 m/s (Digit V–Wrist)	58 m/s (Forearm- Snuff Box	55 m/s Forearm- Snuff Box	48 m/s Calf – lateral malleolus	48 m/s Calf – lateral malleolus	48 m/s Leg – Dorsum of the foot	47 m/s Leg – Dorsum of the foot
Patient 2 (Absent SNAPS) 67Y, Male	Amplitude	8.3 mV	6.3 mV	3.0 mV	NR	NR	NR	NR	NR	NR	NR	NR	NR	NR
	Conduction velocity	54 m/s (Elbow-Wrist)	48 m/s (Below elbow – Wrist) 59 m/s (Above elbow – below elbow)	NC	NA	NA	NA	NA	NA	NA	NA	NA	NA	NA

*NCS* nerve conduction studies, *CMAP* compound muscle action potential, *SNAP* sensory nerve action potential, *ADM* abductor digiti minimi, *R* right, *L* left, *NC* not checked, *NR* not recordable, *NA* not applicable

### Neuroimaging characteristics

All patients had abnormal MRS at diagnosis. Fifteen patients (37.5%) had low NAA/Cr ratio only in the vermis (<0.96) and 6 patients (15%) had low NAA/Cr ratio only in the hemispheres (<1). Nineteen patients (47.5%) had low NAA/Cr ratio in both the vermis and the hemispheres. Only 55% of the patients had cerebellar atrophy in the initial MRI.

### Evidence of autoimmunity

Nineteen patients (47.5%) had gluten sensitivity, of whom 3 patients (7.5%) had biopsy proven coeliac disease. Other abnormal immunological tests were present in another 15 patients (i.e. GAD, ANA etc.), of whom 4 patients (10%) had biopsy proven Sjogren’s syndrome.

### Cancer

Nine patients had a history or developed malignancy (3 bowel, 1 melanoma, 1 light chain myeloma, 2 ovarian, 1 uterus and 1 of unknown primary location). However, only in 6 patients of them the malignancy was diagnosed within 5 years of the neurological symptoms. Anti-Yo antibodies were found in one patient.

### Genetic causes

Nineteen patients were investigated for familial ataxias using next generation sequencing ataxia panel containing 42 genes. In selected patients genetic testing for common mitochondrial mutations (including POLG1) and muscle biopsies were performed. For two of them a genetic cause was identified (one mitochondrial, one SCA18).

### HLA type

HLA typing was performed in 34 patients. Of them, 18 patients (52.9%) had the DQ2 type, 6 patients (17.6%) had the DQ8 type, 9 patients (26.5%) had the DQ1 type and 1 patient (2.9%) had another HLA type.

### Truly idiopathic cases

After extensive immunological and genetic screening, only 3 patients (7.5%) were classified as having a truly idiopathic combination of cerebellar ataxia with SG (Table 2).

**Table 2:** Clinical, neuroimaging and serological characteristics of all patients

Gender	Age at 1st presentation	First symptoms	Age at first symptoms	First diagnosis	Second symptoms	Age at second diagnosis	MRI	Abnormal MRS	SNAPs	Gluten serology	Duodenum biopsy	HLA	Other evidence of autoimmunity	Cancer	Age at cancer diagnosis	Genetics	Truly idiopathic
Female	48	Unsteadiness in the dark	48	SG	Worsening of balance	63	Normal for age	Vermis and hemisphere	Absent	Negative	N/A	DQ2	ENA+ ANA+, Crohn’s			Negative	
Female	59	Patchy numbness	57	SG	No new symptoms	60	Normal for age	Vermis	Asymmetrical	Positive	CD	DQ2	TPO+, dsDNA+, RF+	Ovarian	38		
Male	40	Patchy numbness	38	SG	Worsening of balance	62	Normal for age	Vermis	Absent	Positive	Normal	DQ2	P-ANCA+, ANA+, GM1+, Sarcoidosis				
Male	60	Patchy burning pain	59	SG	No new symptoms	65	Normal for age	Hemisphere	Asymmetrical	Positive	Normal	DQ2					
Female	76	Unsteadiness/Numbness	76	SG	Worsening of balance	84	Normal for age	Hemisphere	Asymmetrical	Positive	CD	DQ2					
Male	49	Patchy numbness	49	SG	No new symptoms	72	Normal for age	Hemisphere	Absent	Negative	N/A	DQ1	ANA+, RF+			Negative	
Male	52	Pain in feet	49	SG	Worsening of balance	70	Normal for age	Vermis	Asymmetrical	Negative	N/A	DQ1	ANA+, P-ANCA+, SACE+				
Female	55	Patchy numbness	55	SG	Worsening of balance	69	Normal for age	Vermis	Asymmetrical	Negative	N/A	Not done	ANA+, ENA+ (Sjogren’s)				
Male	67	Unsteadiness	64	SG	Worsening of balance	68	Cerebellar atrophy	Vermis	Absent	Negative	N/A	DQ2	ANA+, P-ANCA+, MGUS				
Female	61	Patchy pain	61	SG	Worsening of balance	67	Normal for age	Hemisphere	Asymmetrical	Negative	N/A	Not done	ANA+, ENA+ (Sjogren’s)				
Female	58	Patchy numbness	57	SG	Worsening of balance	74	Normal for age	Hemisphere	Asymmetrical	Negative	N/A	Other					Yes
Female	35	Patchy numbness	35	SG	Worsening of balance	49	Normal for age	Vermis and hemisphere	Asymmetrical	Negative	N/A	Not done	ANA+, ENA+ (Sjogren’s)				
Female	42	Unsteadiness	42	Cerebellar ataxia	Patchy sensory loss	46	Cerebellar atrophy	Vermis and hemisphere	Asymmetrical	Negative	N/A	DQ1	Anti-Yo+	Ovarian	41		
Male	14	Unsteadiness	14	Cerebellar ataxia	Worsening of balance	58	Cerebellar atrophy	Vermis	Asymmetrical	Positive	CD	DQ2	GAD+			Mitochondrial	
Female	57	Unsteadiness/Dizziness	55	Cerebellar ataxia	Sensory symptoms	69	Cerebellar atrophy	Vermis	Absent	Positive	Normal	DQ1	GAD+			SCA18	
Male	52	Unsteadiness	51	Cerebellar ataxia	Sensory symptoms	57	Cerebellar atrophy	Vermis and hemisphere	Absent	Positive	Normal	DQ8				Negative	
Female	50	Unsteadiness/ Slurred speech	47	Cerebellar ataxia	Sensory symptoms	54	Cerebellar atrophy	Vermis and hemisphere	Asymmetrical	Positive	Not done	DQ2	RF+			Negative	
Male	44	Unsteadiness	39	Cerebellar ataxia	No new symptoms	46	Cerebellar atrophy	Vermis	Asymmetrical	Positive	Normal	DQ8				Negative	
Female	54	Unsteadiness	53	Cerebellar ataxia	Sensory symptoms	60	Cerebellar atrophy	Vermis and hemisphere	Asymmetrica	Positive	Normal	DQ2		Colon	69	Negative	
Male	67	Unsteadiness	54	Cerebellar ataxia	No new symptoms	76	Cerebellar atrophy	Vermis and hemisphere	Absent	Positive	Normal	DQ2	ANA+			Negative	
Male	66	Unsteadiness	66	Cerebellar ataxia	No new symptoms	67	Cerebellar atrophy	Vermis and hemisphere	Absent	Negative	N/A	DQ2	ANA+	Bowel	68		
Female	66	Unsteadiness	63	Cerebellar ataxia	Sensory symptoms	68	Normal for age	Vermis and hemisphere	Absent	Negative	N/A	DQ2				Negative	Yes
Female	65	Unsteadiness	59	Cerebellar ataxia	Patch sensory loss	68	Cerebellar atrophy	Vermis	Absent	Negative	N/A	DQ8	C-ANCA+, MGUS	Myltiple myeloma - light chain	70	Negative	
Female	46	Unsteadiness	46	Cerebellar ataxia	Patchy sensory loss	58	Cerebellar atrophy	Vermis	Asymmetrical	Negative	N/A	DQ1	TPO+			Negative	
Female	66	Unsteadiness	64	Cerebellar ataxia	No new symptoms	69	Normal for age	Vermis and hemisphere	Asymmetrical	Negative	N/A	DQ2				Negative	Yes
Female	68	Unsteadiness	67	Cerebellar ataxia	Sensory symptoms	69	Normal for age	Vermis and hemisphere	Absent	Negative	N/A	DQ2	ANA+, TPO+				
Male	81	Unsteadiness	79	Cerebellar ataxia	Worsening of balance	85	Normal for age	Vermis and hemisphere	Asymmetrical	Negative	N/A	Not done	MGUS	Melanoma/ Possibly prostate	86		
Female	29	Unsteadiness	8	Cerebellar ataxia	Sensory symptoms	33	Cerebellar atrophy	Vermis and hemisphere	Asymmetrical	Positive	Normal	DQ2	OCB+, TPO+, GAD+ (one occasion only)			Negative	
Male	43	Unsteadiness	34	Both at the same time		43	Cerebellar atrophy	Hemisphere	Asymmetrical	Positive	Normal	DQ2	RF+				
Female	61	Unsteadiness/Numbness	60	Both at the same time		61	Cerebellar atrophy	Vermis	Absent	Positive	Normal	DQ1		Malignancy of unknown primary location	64	Negative	
Female	81	Unsteadiness	80	Both at the same time		81	Cerebellar atrophy	Vermis	Asymmetrical	Positive	Normal	Not done					
Male	51	Unsteadiness/Numbness	51	Both at the same time		51	Cerebellar atrophy	Vermis and hemisphere	Asymmetrical	Positive	Normal	DQ1					
Female	55	Unsteadiness/Pain	49	Both at the same time		55	Cerebellar atrophy	Vermis	Absent	Negative	N/A	DQ1	GAD+, P-ANCA+	Uterus	39	Negative	
Male	64	Unsteadiness	54	Both at the same time		64	Cerebellar atrophy	Vermis and hemisphere	Absent	Negative	N/A	DQ8	MGUS			Negative	
Male	70	Unsteadiness	67	Both at the same time		70	Normal for age	Vermis	Absent	Negative	N/A	DQ1	C-ANCA+				
Male	54	Unsteadiness	49	Both at the same time		54	Cerebellar atrophy	Vermis and hemisphere	Asymmetrical	Negative	N/A	DQ2	TPO+				
Female	70	Unsteadiness	69	Both at the same time		70	Normal for age	Vermis and hemisphere	Asymmetrical	Negative	N/A	Not done	ANA+, ENA+ (Sjogren’s), RF+	Bowel	71		
Male	57	Unsteadiness	55	Both at the same time		57	Cerebellar atrophy	Vermis	Absent	Positive	Normal	DQ2				Negative	
Male	61	Unsteadiness/Numbness	61	Both at the same time		62	Normal for age	Vermis and hemisphere	Asymmetrical	Positive	Normal	DQ8	ENA+				
Female	69	Unsteadiness	64	Both at the same time		69	Cerebellar atrophy	Vermis and hemisphere	Asymmetrical	Positive	Normal	DQ8	P-ANCA+				

## Discussion

Our case series highlights that amongst patients with cerebellar ataxia and sensory neuronopathy, where a genetic or a malignant cause is not identified, immune pathogenesis is a likely cause. In fact out of 40 patients, only 2 were found to have a genetic cause and only 6 patients had malignancy, which was diagnosed within 5 years of their symptoms. From the remaining 32 patients the vast majority (91%) had evidence of autoimmunity and only 3 patients (9%) were classified as truly idiopathic.

Whether considering the cut-off of 5 years for a neurological syndrome to be classified as paraneoplastic is a matter of debate [11]. This time period has been based on reports showing that in the majority of cases the interval between the paraneoplastic neurological syndrome and the diagnosis of malignancy is less than 5 years [11]. However in our case series, there are patients who exceed this interval. The type of malignancy, however, may influence this interval.

The HLA type has been linked to predisposition to autoimmunity. In our case series 70.5% of patients had the DQ2 or the DQ8 HLA subtypes, which are known to be associated with autoimmunity. This percentage is significantly higher compared to the 40% of the general population [12]. In addition, in our cohort, evidence for autoimmunity (serological evidence of gluten sensitivity and/or positive other antibody titers) was present in 90% of the patients.

Our findings should be interpreted with some caution given the limitations of our design. Firstly, as this is a retrospective observational case series of patients regularly attending our Ataxia clinic not all patient’s had full genetic screening, which means that the percentage of genetic causes might be higher than the one we reported. However, the bias is probably minimized by the fact that we genetically tested patients with early onset and/or family history of ataxia and SG. When clinically suspected, patients also underwent genetic testing for mitochondrial diseases including muscle biopsies. Despite this it is possible that in some patients mitochondrial aetiology [13, 14] may have been missed. Secondly, we haven’t routinely been examining for the vestibulo-ocular reflex and therefore cases of CANVAS may have been missed. However, our understanding is that most cases with CANVAS, unlike the series here, tend to have a family history.

Both cerebellar ataxia (gluten ataxia) and sensory ganglionopathy in isolation have been previously linked to gluten sensitivity [4, 10, 15]. Almost half of the patients in this report (47.5%) had serological evidence of gluten sensitivity without enteropathy (coeliac disease). Gluten free diet has already been shown to be beneficial in such patients [4] and should be considered in all patients with cerebellar ataxia and sensory ganglionopathy in the presence of serological markers of sensitivity to gluten.
